# Inflammatory Effects and Regulatory Mechanisms of Chitinase-3-like-1 in Multiple Human Body Systems: A Comprehensive Review

**DOI:** 10.3390/ijms252413437

**Published:** 2024-12-15

**Authors:** Dong Liu, Xin Hu, Xiao Ding, Ming Li, Lei Ding

**Affiliations:** 1 School of Life Sciences, Yunnan University, Kunming 650500, China; liudong@ynu.edu.cn; 2 Yunnan Key Laboratory of Soil Erosion Prevention and Green Development, Institute of International Rivers and Ecosecurity, Yunnan University, Kunming 650500, China; huxin@itc.ynu.edu.cn; 3 Key Laboratory of Phytochemistry and Natural Medicines, Kunming Institute of Botany, Chinese Academy of Sciences, Kunming 650201, China; dingxiao@mail.kib.ac.cn

**Keywords:** chitinase-3-like-1, inflammatory diseases, human body systems, regulatory mechanisms, biomarkers

## Abstract

Chitinase-3-like-1 (Chi3l1), also known as YKL-40 or BRP-39, is a highly conserved mammalian chitinase with a chitin-binding ability but no chitinase enzymatic activity. Chi3l1 is secreted by various cell types and induced by several inflammatory cytokines. It can mediate a series of cell biological processes, such as proliferation, apoptosis, migration, differentiation, and polarization. Accumulating evidence has verified that Chi3l1 is involved in diverse inflammatory conditions; however, a systematic and comprehensive understanding of the roles and mechanisms of Chi3l1 in almost all human body system-related inflammatory diseases is still lacking. The human body consists of ten organ systems, which are combinations of multiple organs that perform one or more physiological functions. Abnormalities in these human systems can trigger a series of inflammatory environments, posing serious threats to the quality of life and lifespan of humans. Therefore, exploring novel and reliable biomarkers for these diseases is highly important, with Chi3l1 being one such parameter because of its physiological and pathophysiological roles in the development of multiple inflammatory diseases. Reportedly, Chi3l1 plays an important role in diagnosing and determining disease activity/severity/prognosis related to multiple human body system inflammation disorders. Additionally, many studies have revealed the influencing factors and regulatory mechanisms (e.g., the ERK and MAPK pathways) of Chi3l1 in these inflammatory conditions, identifying potential novel therapeutic targets for these diseases. In this review, we comprehensively summarize the potential roles and underlying mechanisms of Chi3l1 in inflammatory disorders of the respiratory, digestive, circulatory, nervous, urinary, endocrine, skeletal, muscular, and reproductive systems, which provides a more systematic understanding of Chi3l1 in multiple human body system-related inflammatory diseases. Moreover, this article summarizes potential therapeutic strategies for inflammatory diseases in these systems on the basis of the revealed roles and mechanisms mediated by Chi3l1.

## 1. Introduction

The human body is composed of ten main organ systems, including the digestive, respiratory, nervous and sensory, circulatory, endocrine, lymphatic, muscular, skeletal, reproductive, and urinary systems [1,2]. Specifically, the digestive system includes the digestive gland and digestive tract and functions in nutrient absorption and waste excretion; the respiratory system performs the function of gas exchange, which relies on the nose, pharynx, larynx, trachea, bronchus, and lungs; the nervous system mainly includes the central nervous system and the peripheral nervous system to guarantee the normal activity of the human body; the circulatory system includes the heart, arteries, veins, blood, etc.; the endocrine system mainly includes endocrine glands and tissues, which control and regulate organisms by secreting special chemicals; the lymphatic system mainly includes lymphatic vessels and lymphatic organs; the muscular and skeletal systems play a role in motion, support, and protection of the human body through the organs of bone, joints, and skeletal muscles; the reproductive system mainly consists of the genital gland, genital tract, and accessory organs, which function in reproducing offspring; and the urinary system excretes various metabolic substances through the kidney, bladder, ureter, and urethra. Each organ system is closely correlated with human health [2,3]. Exploring reliable diagnostic biomarkers for the progression of human body system-related inflammatory diseases, especially in the early stages of disease, is highly important. Chi3l1 can be used to effectively diagnose and determine disease activity, severity, and prognosis across multiple stages of disease. Over the past three decades, serum Chi3l1 (known as YKL-40 or BRP-39) has been extensively reported as a critical parameter for various inflammatory diseases related to human organ systems [4,5].

As a member of the glycoside hydrolase family 18, Chi3l1 acts as a conserved and secreted glycoprotein with chitin-binding ability but without chitinase enzymatic activity. The structure of Chi3l1 suggests that it functions as a sensor to initiate innate defenses and regulate inflammatory responses. Chi3l1 can be expressed in multiple cell types, such as macrophages, neutrophils, epithelial cells, astrocytes, monocytes, stem cells, synoviocytes, chondrocytes, and smooth muscle cells [6,7,8,9,10,11,12,13,14]. Accumulating evidence has shown that the expression of Chi3l1 can be regulated by several inflammatory cytokines, such as interleukin-6 (IL-6) and interferon-γ (IFN-γ); however, Chi3l1 can also regulate the transcriptional expression of some inflammatory cytokines to modulate the immune response and tissue repair in inflammatory diseases [5,15]. Chi3l1 has potential functions in cell proliferation, apoptosis, migration, differentiation, polarization, and activation through binding to its receptors (e.g., interleukin 13 receptor alpha 2 (IL-13Rα2) and syndecan-1/αVβ3) [5,16,17,18,19,20,21]. Chi3l1 is extensively involved in all human body system-related inflammatory diseases. It can serve as an effective diagnostic biomarker and can be used to evaluate disease activity, severity, and prognosis. As a systematic and comprehensive understanding of the inflammatory effects and regulatory mechanisms of Chi3l1 on the whole organ system is lacking, a summary of this scientific question will contribute to understanding inflammatory diseases involving Chi3l1 in specific organ systems.

In this review, we comprehensively summarize Chi3l1-mediated inflammatory diseases involving almost all human body systems and organs on the basis of recent reports. The potential roles and underlying mechanisms of Chi3l1 in various inflammatory environments are discussed, and potential therapeutic strategies are also outlined.

## 2. Roles and Mechanisms of Chi3l1 in Respiratory System Diseases

### 2.1. Lung

**Figure 1:**
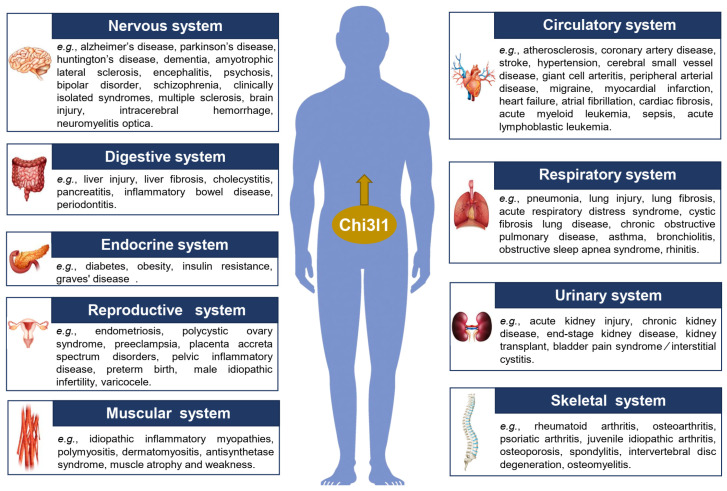
Summary of inflammatory diseases associated with elevated levels of Chi3l1 in various human body systems. Chi3l1 is highly expressed and acts as a valuable biomarker in inflammatory conditions affecting the nervous, digestive, endocrine, reproductive, muscular, circulatory, respiratory, urinary, and skeletal systems. The arrow indicates the increased Chi3l1 levels.

Many studies have reported that Chi3l1 serves as a useful biomarker for assessing the severity and prognosis of multiple inflammatory pulmonary diseases, including pneumonia, lung injury, and lung fibrosis (Figure 1).

Pneumonia, a common acute respiratory infection, is closely related to Chi3l1 levels [22]. Community-acquired pneumonia (CAP) is a typical acute pulmonary disease, and serum levels of Chi3l1 dramatically increase in patients with CAP [23]. However, Chi3l1 levels are significantly correlated with disease severity and prognosis in CAP patients infected by viruses but not bacteria, which is verified by the negative correlation between the reduction degree of Chi3l1 levels for the disease severity and the median length of hospital stay in the viral pneumonia group rather than in the bacterial pneumonia group [24]. Hypersensitivity pneumonitis (HP) patients, exposed repeatedly to organic particles, present relatively high levels of Chi3l1 in their serum [25,26]. Moreover, there is also an observed correlation between Chi3l1 serum levels and the diffusing capacity of the lung for carbon monoxide in these patients [25].

**Figure 2:**
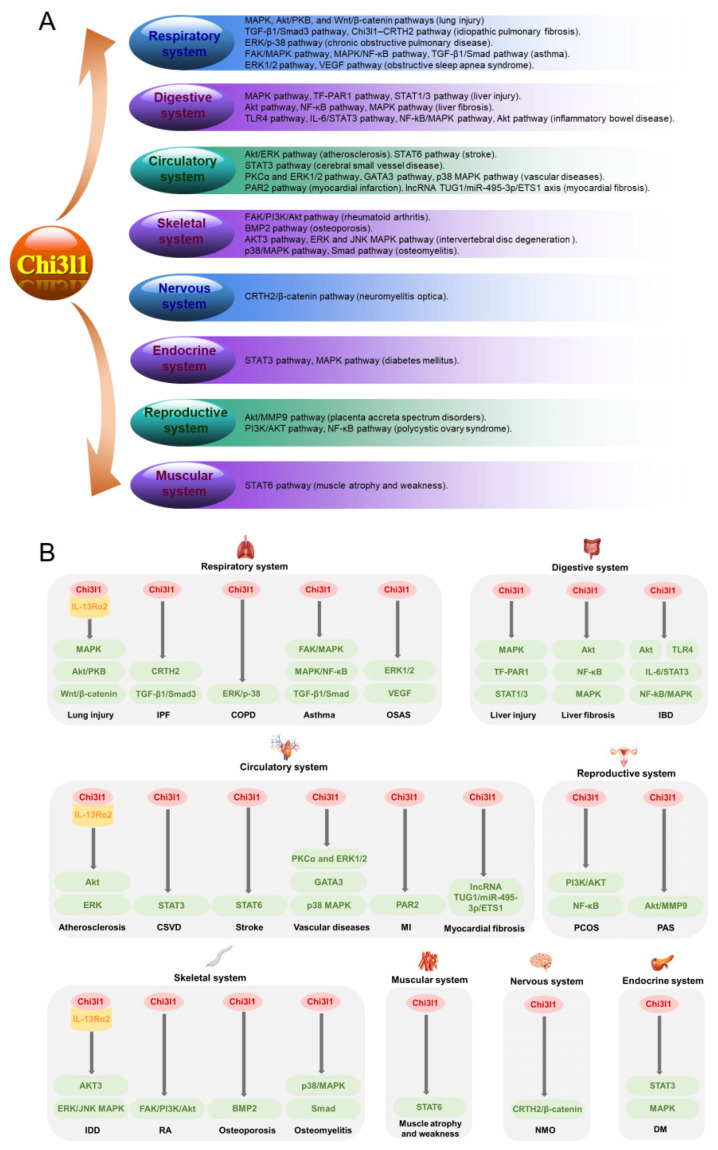
Summary of the regulatory mechanisms by which Chi3l1 is involved in multiple human body system diseases. Various signaling pathways are involved in the regulation of Chi3l1 upregulation-related inflammatory diseases. The cascades associated with specific diseases of human organ systems (e.g., respiratory, digestive, circulatory, skeletal, nervous, endocrine, reproductive, and muscular systems) are shown in (**A**), and the detailed diagrams are shown in (**B**).

Lung injury and fibrosis are also classified as respiratory system diseases, and Chi3l1 levels are closely related to the pathogenesis of these diseases. Lung injury can be induced by various factors, such as hyperoxia, oxidants, and viruses [21,27,28]. Chi3l1 contributes to inhibiting hyperoxic acute lung injury (HALI) and prolonging mouse survival in 100% O_2_, and it also alleviates oxidant-induced lung injury by binding to the IL-13Rα2 receptor [21,27]. Mechanistically, Chi3l1 can bind to IL-13Rα2 and activate MAPK, Akt/PKB, and Wnt/β-catenin signaling [21]. Chi3l1 levels can reflect the extent of lung injury in COVID-19 patients. Further mechanistic studies have verified that Chi3l1 can stimulate the expression and accumulation of the virus receptor angiotensin-converting enzyme 2 (ACE2) and viral spike protein priming protease (SPP) to augment viral infection [28,29]. Acute respiratory distress syndrome (ARDS) is a multifactorial syndrome that may cause acute lung injury. The knockdown of *Chi3l1* can suppress the inflammatory response and apoptosis in an in vitro model of ARDS [30]. Pulmonary fibrosis is a general term for fibrotic lung disorders, and idiopathic pulmonary fibrosis (IPF) is the most common and particularly deadly form of pulmonary fibrosis, with a median survival of only 3 years [31]. Serum Chi3l1 levels are highly elevated in patients with IPF. Chi3l1 can ameliorate inflammation and cell death to play a protective role during the injury phase and augment alternative macrophage activation, fibroblast proliferation, and matrix deposition to play a profibrotic role during the repair phase [32]. Furthermore, Chi3l1 may promote the progression of IPF and interstitial transformation of alveolar epithelial cells through the TGF-β1/Smad3 signaling pathway, and the Chi3l1–CRTH2 pathway functions in regulating monocyte/macrophage responses in patients with IPF (Figure 2) [33,34]. As a severe autosomal genetic disease, cystic fibrosis (CF) generally affects many organs. However, CF lung disease primarily manifests as an infectious disorder characterized by progressively declining lung function [35,36]. Reportedly, elevated Chi3l1 levels are strongly linked with CF pathology in both pediatric and adult patients with CF-related lung disease, and neutrophils are considered a potential source of increased Chi3l1 [35,36].

### 2.2. Respiratory Tract

The respiratory tract functions as a passage for gas, and its associated inflammatory diseases (e.g., chronic obstructive pulmonary disease, asthma, bronchiolitis, obstructive sleep apnea syndrome, and rhinitis) are also related to increased serum levels of Chi3l1 (Figure 1). Chi3l1 reportedly mediates the secretion of the inflammatory cytokine IL-8 via MAPK pathways in airway epithelial cells [6].

Chronic obstructive pulmonary disease (COPD), a significant global health issue, is characterized mainly by chronic airway inflammation and increasing rates of mortality and morbidity. Numerous studies have shown elevated serum levels of Chi3l1 in patients with COPD, indicating a significant relationship between high Chi3l1 levels and increased mortality among COPD patients [37]. Recent studies have reported that high Chi3l1 levels are associated with an increased neutrophil percentage and neutrophil-to-lymphocyte ratio, as well as lower lymphocyte-, eosinophil-, and basophil-related parameters [38,39]. However, they are not significantly correlated with airflow obstruction, dyspnea, exercise capacity (BODE) index, O_2_ saturation, or smoking index in patients with COPD [38,39]. Chi3l1 is predominantly secreted by proinflammatory MΦ1 cells rather than by anti-inflammatory MΦ2 cells in COPD. It has a potential role in remodeling the airway by increasing collagen production from lung fibroblasts via the ERK and p38 pathways, as well as activating alveolar macrophages with increased production of IL-8, TGFβ, MCP-1, MIP-1α, and MMP-9 (Figure 2) [40,41,42].

Asthma is a common chronic inflammatory respiratory disease characterized by chronic airway inflammation, bronchial hyperresponsiveness, and reversible airway obstruction. A significant amount of evidence has revealed a close correlation between serum Chi3l1 levels and the diagnosis and severity of asthma [43]. Recent research further suggests that Chi3l1 can be differentially expressed in various respiratory tract diseases, such as asthma, COPD, and asthma–COPD overlap syndrome (ACO) and that it functions in predicting the loss of lung function and neutrophilic airway inflammation in patients with these diseases [44,45]. Single nucleotide polymorphisms (SNPs) in the *Chi3l1* promoter effectively affect the airway expression of Chi3l1 and the severity of asthma. Various polymorphisms of Chi3l1, including rs4950928, rs10399931, rs883125, and rs12141494, are important genetic factors for asthma [46,47]. In addition, specific subgroups of Chi3l1 are associated with the severity of asthma. Among them, Chi3l1 cluster 3 (C3) harbors common features of severe airflow obstruction, earlier onset, and longer duration of disease, near-fatal asthma exacerbations, and upregulation of the NLRP3 inflammasome and microRNA 223 [48]. Chi3l1 is reported to be a non-type 2 inflammatory signature for non-eosinophilic asthma (NEA), which is correlated with inflammatory phenotypes, anti-asthma responsiveness, and future exacerbations [49]. In an ovalbumin (OVA)-induced murine model of acute asthma, Chi3l1 contributes to augmenting the Th2 inflammatory response and airway hyperresponsiveness, as well as promoting dendritic cell maturation [50]. Mechanistically, Chi3l1 levels are significantly correlated with airway remodeling and lung function, which are mediated by the activation of the FAK and MAPK signaling pathways (Figure 2) [51,52,53]. Chi3l1 potentially promotes bronchial smooth muscle proliferation and migration by inducing IL-8 expression in the bronchial epithelium via the MAPK and NF-κB pathways in asthma (Figure 2) [54]. Additionally, Chi3l1 can regulate the proliferation, apoptosis, and migration of human bronchial epithelial cells via the TGF-β1/Smad cascade (Figure 2) [17].

Bronchiolitis, obstructive sleep apnea syndrome, and rhinitis are also common inflammatory diseases of the respiratory tract. Serum Chi3l1 levels act as a critical biomarker for bronchiolitis obliterans, which further contributes to distinguishing exacerbation of postinfectious bronchiolitis obliterans from acute bronchiolitis in young children [55,56]. Additionally, Chi3l1 levels can be used to diagnose and assess the severity of obstructive sleep apnea syndrome (OSAS), with the concentrations of Chi3l1 in serum showing better diagnostic capabilities for moderate and severe OSAS than those in plasma [57,58]. Chi3l1 may play an important role in the pathogenesis of OSAS by increasing the proliferation of tonsil lymphocytes via the ERK1/2 pathway and causing endothelial dysfunction via VEGF signaling, thereby potentially triggering or exacerbating many serious complications, such as hypertension and liver fibrosis in patients with OSAS (Figure 2) [59,60,61,62]. Increased serum levels of Chi3l1 are also detected in patients with rhinitis, such as moderate/severe persistent allergic rhinitis (M/S PAR) and chronic rhinosinusitis with nasal polyps (CRSwNP), possibly contributing to remodeling the nasal mucosa, distinguishing CRSwNP endotypes, and predicting postoperative recurrence [63,64].

## 3. Roles and Mechanisms of Chi3l1 in Diseases of the Digestive System

### 3.1. Digestive Gland

The digestive gland serves as a vital biological component of the digestive system, the abnormality of which seriously threatens human health. Many studies have verified that Chi3l1 can be used to diagnose and evaluate digestive gland-associated inflammatory diseases such as liver injury, liver fibrosis, cholecystitis, and pancreatitis (Figure 1).

Liver injury can be caused by multiple factors, such as drug toxicity and alcohol abuse, and is characterized by ubiquitous hepatotoxicity and hepatocellular necrosis [65,66]. Hepatic CD14^+^ cells are an important source of Chi3l1 mRNA and protein in liver injury, with expression patterns similar to those of growth factors implicated in inflammation fibrogenesis [67]. In lipopolysaccharide (LPS)-induced liver injury, deficiency or inhibition of Chi3l1 expression contributes to ameliorating disease progression by inhibiting M2 macrophage polarization or downregulating the chemokine CXCL3, respectively [68,69]. The activation of tissue factor (TF) can induce intrahepatic vascular coagulation and, eventually, lead to liver injury. In a mouse model of concanavalin A (ConA)-induced liver injury, Chi3l1 exacerbates disease progression by inducing TF expression via MAPK activation and the subsequent enhancement of intrahepatic coagulation activation, as well as by inducing chemokine ligand 2 (CCL2) and IP-10 expression via activating the TF-PAR1 pathway and the subsequent increase in inflammatory cells recruitment (Figure 2) [70,71]. In thioacetamide (TAA)-induced liver injury, Chi3l1 potentially alleviates liver damage by reducing IFN-γ expression and inhibiting Th1 cell differentiation via the STAT3 signaling pathway (Figure 2) [66]. In ethanol-induced liver injury, Chi3l1 deficiency contributes to attenuating disease severity by inhibiting sterol regulatory element binding protein 1 (SREBP1)-dependent triglyceride synthesis [72]. In hepatic ischemia-reperfusion (HIR) injury, MMP activation and Chi3l1 upregulation likely result in profibrotic and proinflammatory cytokine release [73]. Additionally, Chi3l1 secreted by mesenchymal stem cells (MSCs) can effectively suppress STAT1/3 signaling in T cells through the upregulation of peroxisome proliferator-activated receptor δ (PPARδ), thereby alleviating immune-mediated liver injury (Figure 2) [74].

Liver fibrosis can be described as a chronic liver inflammatory disease with common features of hepatic stellate cell activation and excess collagen deposition caused by various factors, such as virus infection, liquor, and toxins [75]. Accumulating evidence has demonstrated that serum Chi3l1 levels tend to increase with the progression of liver fibrosis; thus, Chi3l1 is a noninvasive serum biomarker for diagnosing and staging liver fibrosis [75,76]. Mechanistically, Chi3l1 can exacerbate the progression of liver fibrosis by inhibiting hepatic macrophage apoptosis through the suppression of Fas expression and the activation of Akt signaling (Figure 2) [77]. Chi3l1 has diagnostic value for liver fibrosis diseases of different backgrounds, including metabolic-associated diseases (e.g., nonalcoholic fatty liver disease (NAFLD), nonalcoholic steatosis disease (NASH), and alcoholic liver disease (ALD)) and infection-associated diseases (e.g., hepatitis B virus (HBV)-related and hepatitis C virus (HCV)-related liver fibrosis) [75]. In NAFLD, macrophage-derived Chi3l1 levels increase in accordance with the progression of liver fibrosis [78]. Additionally, Chi3l1 can markedly blunt hepatic insulin signaling as measured by reduced pAKT, pGSK-3β, and pERK levels in NAFLD, suggesting that Chi3l1 may play a role in the development of hepatic insulin resistance associated with inflammation and lipid deposition [79]. As a progressive form of NAFLD, NASH progression can be affected by Chi3l1 by regulating the NLRP3 inflammasome and influencing the cellular activation, recruitment, and infiltration of macrophages and neutrophils [80]. In a NASH mouse model, Chi3l1 has also been shown to be associated with disease progression through regulating fibrosis-promoting factors via macrophages and directly activating hepatic stellate cells (HSCs) via the receptor IL13Rα2 [81]. In ALD patients, high levels of Chi3l1 can reflect the severity and remodeling of liver fibrosis [82]. In HBV-related liver diseases, Chi3l1 not only effectively reflects liver fibrosis severity before antiviral therapy but can also be used to monitor changes in liver fibrosis during therapy [83]. Most patients with HBeAg-negative chronic hepatitis B have an elevated risk of cirrhosis and liver cancer, and serum Chi3l1 can act as a diagnostic marker and risk factor for liver fibrosis in these patients [84]. In HCV-related liver diseases, Chi3l1 is a promising marker for estimating the degree of liver fibrosis and evaluating the efficacy of IFN therapies [85]. Moreover, fibrosis progression seems to be under genetic control, and the Chi3l1-131G → C promoter polymorphism is reported to be associated with the severity of HCV-induced liver fibrosis through the determination of Chi3l1 serum levels [86]. Mechanistically, HCV induces the secretion of Chi3l1 in hepatic parenchymal cells by activating nuclear factor-κB (NF-κB)-dependent pathways via cooperative induction of the tumor necrosis factor-α (TNF-α) and reactive oxygen species (ROS)-mitogen-activated protein kinase (MAPK) pathways, thereby further enhancing the progression of liver fibrosis via the interaction between HCV and Chi3l1 (Figure 2) [87].

Pancreatitis and cholecystitis are also common digestive gland-related inflammatory diseases. Elevated serum Chi3l1 levels can be used for diagnosing pancreatitis, and macrophages may be involved in the pancreatic microenvironment during disease progression [88]. A recent study suggested that Chi3l1 is also a novel and effective biomarker for the diagnosis of acute cholecystitis [89]. However, further research is needed to understand the underlying mechanisms of Chi3l1 in these two types of diseases.

### 3.2. Digestive Tract

Some digestive tract-associated chronic inflammatory diseases, such as inflammatory bowel disease (IBD) and periodontitis, are difficult to cure and pose severe threats to human health. Chi3l1 is reported to be an effective diagnostic biomarker and a promising therapeutic target for these diseases (Figure 1).

Inflammatory bowel disease (IBD), characterized by chronic and progressive disorders of the gastrointestinal tract, can be classified into two main subtypes: Crohn’s disease (CD) and ulcerative colitis (UC). Both serum and fecal Chi3l1 levels are reliable biomarkers in pediatric and adult patients with IBD [90,91,92]. Chi3l1 is expressed by intestinal epithelial cells (IECs) and macrophages in the inflamed intestines of both CD patients and UC patients, but not in those of healthy controls [93]. Specifically, the Chi3l1 protein seems to be produced on the apical sides of colonic epithelial cells, mainly in the active regions of CD patients [93]. Furthermore, Chi3l1 potentially plays a role in the proliferation and migration progression of IECs through crosstalk between Chi3l1-mediated intracellular signaling cascades and TLR4 signaling, as well as through regulating the proapoptotic S100A9 protein (Figure 2) [94,95]. Depending on the chitin-binding motif, Chi3l1 also activates Akt signaling in colonic epithelial cells, possibly related to the development of chronic colitis (Figure 2) [96]. Chronic bacterial infections are reportedly involved in the pathogenesis of IBD, while Chi3l1 can exacerbate intestinal inflammation by enhancing bacterial adhesion and invasion through interactions with bacterial chitin-binding proteins in IECs [93,97]. In addition, Chi3l1 is required for mediating selected Gram-negative bacterial infectious colitis; Chi3l1 and IL-6, but not IL-22, synergistically activate the STAT3 signaling pathway in IECs, which is associated with the activation of the NF-kB and MAPK pathways (Figure 2) [98]. Recent studies suggest that Chi3l1 is a novel neutrophil autoantigenic target in IBD, whereas IgA and secretory IgA (sIgA) to Chi3l1 may facilitate the serological diagnosis of IBD [99,100].

Periodontitis can be classified as a digestive system-associated inflammatory disease, considering the importance of the oral cavity in the digestive tract. In recent years, Chi3l1 in serum and gingival crevicular fluid has been identified as a novel biomarker for diagnosing and assessing the severity of periodontitis, whereas nonsurgical periodontal therapy and *Moringa oleifera* can effectively decrease Chi3l1 levels in chronic periodontitis patients or a periodontitis rat model [101,102,103].

## 4. Roles and Mechanisms of Chi3l1 in Diseases of the Circulatory System

### 4.1. Blood Vessels

Many studies have demonstrated that Chi3l1 is a reliable biomarker and promising therapeutic target for blood vessel-related inflammatory diseases such as atherosclerosis, coronary artery disease, stroke, and hypertension (Figure 1) [104].

Atherosclerosis is a chronic inflammatory disease, and Chi3l1 is overexpressed in patients with atherosclerosis [105,106]. Chi3l1 can predict plaque instability, potentially reflecting macrophage activation and matrix degradation within atherosclerotic lesions [106]. This is supported by the ability of Chi3l1 to induce the release of “pro-atherogenic” chemokines in macrophages and the activation of matrix metalloproteinase-9 in THP-1 monocytes [106]. Moreover, Chi3l1 can exacerbate atherosclerosis by mediating endothelial cell (EC) inflammation and vascular smooth muscle cell (VSMC) activation [107]. Mechanistically, Chi3l1 inhibits macrophage apoptosis by upregulating Aven to suppress the activation of caspase-9 in early-stage atherosclerosis (Figure 2) [108]. The ERK and AKT signaling pathways are the main molecular adjusting networks involved in promoting the proliferation of HUVECs, and Chi3l1 can promote angiogenic formation by interacting with interleukin-13 receptor α2 (IL-13Rα2) via these two pathways in late-stage atherosclerotic lesions (Figure 2) [109]. However, Chi3l1 reportedly contributes to the amelioration of LPS-induced atherosclerotic reactions via PPARδ-mediated suppression of inflammation and endoplasmic reticulum stress [110].

Coronary artery disease (CAD) is one of the most frequently occurring diseases related to vascular inflammation. Numerous studies have shown that serum Chil3l1 can serve as a useful biomarker for the diagnosis and severity of CAD independent of common risk factors plus high-sensitivity C-reactive protein (hs-CRP) and N-terminal-pro-B natriuretic peptide (NT-proBNP), whereas treatment with certain drugs, such as statins, has no influence on the effectiveness of Chi3l1 as a superior prognostic biomarker in CAD patients [111,112,113]. Chi3l1 levels significantly increase and are associated with myocardial injury and leukocyte-activating factors following coronary artery bypass surgery; thus, it acts as a potential indicator of myocardial injury and subsequent fibrosis after heart surgery [114]. Additionally, Chi3l1 genetic polymorphisms are potentially associated with CAD [115].

Stroke is regarded as a major cause of death and disability worldwide. Accumulating evidence has demonstrated that serum Chi3l1 levels are significantly correlated with infarct volume, stroke severity, and functional outcome in acute ischemic stroke (AIS) patients, whereas increased Chi3l1 levels appear to be associated with periventricular white matter hyperintensity (PV-WMH) but not overall WMH or deep WMH (D-WMH) in AIS patients [116,117,118]. Reportedly, Chi3l1 is significantly associated with worse outcomes in acute ischemic stroke patients [117]. Additionally, single nucleotide polymorphisms (SNPs) of Chi3l1 (e.g., rs872129) are correlated with an increased risk of mortality from ischemic stroke [119]. According to previous reports, Chi3l1 deficiency contributes to stroke development by enhancing neuroinflammation through decreasing STAT6-dependent M2 macrophage polarization (Figure 2) [120].

Hypertension is a common cardiovascular disease (CVD) with a potential risk of macrovascular events by increasing arterial wall stiffness. Hypertension patients exhibit high Chi3l1 levels, which have been demonstrated to be associated with increased arterial stiffness, modifiable risk factors for CVD, major cardiovascular outcomes, endothelial dysfunction, and inflammation [121,122,123,124]. According to a nested case-control study in China, Chi3l1 is correlated with hypertension incidence only among men but not women, and it also predicts the risk of developing hypertension in the prehypertensive population [125,126]. Reportedly, the genetic variant for rs10399805 is associated with higher Chi3l1 levels, whereas the genetic variants for rs2297839 and rs4950928 have lower Chi3l1 levels; however, all three SNPs of Chi3l1 could significantly improve the accuracy of risk prediction for hypertension [127].

Other blood vessel-related inflammatory diseases can also be evaluated with the Chi3l1 biomarker. For example, elevated serum levels of Chi3l1 have been identified as novel biomarkers for cerebral small vessel disease (CSVD) [128,129]. The disruption of white matter macrostructure and microstructure is significantly associated with increased Chi3l1 levels, whereas white matter damage can mediate the associations between increased serum Chi3l1 levels and cognitive impairment [128]. The adenosine A2a receptor can suppress astrocyte-mediated inflammation through the inhibition of the STAT3/Chi3l1 axis and, thereby, reduce white matter damage in CSVD patients (Figure 2) [130]. Giant cell arteritis (GCA) is the most common inflammatory disease of medium and large arteries, and the CD206+MMP-9+ macrophage subset mediates tissue destruction and neovascularization through the Chi3l1/IL-13Rα2 axis in this disease [131]. Additionally, increased Chi3l1 levels are independently associated with poor long-term cardiovascular survival in peripheral arterial disease patients [132]. Migraine is a neurovascular disorder, and serum Chi3l1 levels are significantly higher in migraine patients than in controls [133]. The elevation of Chi3l1 levels in migraine patients suggests the presence of neurovascular inflammation in the pathogenesis of migraine [133]. Mechanistically, syndecan-4 can mediate the effects of Chi3l1 on the migration and tube formation of human umbilical vein cells (HUVECs) through the PKCα and ERK1/2 signaling pathways (Figure 2) [134]. Chi3l1 knockdown exacerbates vascular smooth muscle cell (VSMC) phenotypic switching and worsens disease outcomes by increasing CD68 expression, promoting cell proliferation, and inducing cell apoptosis [135]. The zinc-finger transcription factor GATA3 and p38 MAPK are involved in regulating cellular responses such as cell proliferation, growth, differentiation, migration, and apoptosis. GATA3 can downregulate vascular endothelial growth factor (VEGF) and contribute to regulating endothelial cells’ inhibited function [136]. Reportedly, *Helicobacter pylori* infection contributes to vascular endothelial injury by inducing GATA3-dependent Chi3l1 upregulation and activating the p38 MAPK pathway (Figure 2) [136].

### 4.2. Heart

The heart acts as a central component of the circulatory system. Its related diseases, such as myocardial infarction, heart failure, atrial fibrillation, and cardiac fibrosis, remain one of the leading causes of death globally. In patients with myocardial infarction (MI), serum Chi3l1 levels greatly increase, which correlates with the levels of C-reactive protein, matrix metalloproteinase-9, and brain natriuretic protein; diastolic dysfunction; and long-term increased overall mortality [137,138]. Moreover, postinfarction exercise training can improve myocardial function and enhance cardiac angiogenesis by activating Chi3l1/PAR2 signaling (Figure 2) [139]. In addition, elevated Chi3l1 levels are detected in patients with heart failure [140]. As a diagnostic biomarker for cardiac fibrosis, Chi3l1 can promote myocardial fibrosis by regulating the lncRNA TUG1/miR-495-3p/ETS1 axis (Figure 2) [141,142]. In patients with atrial fibrillation, Chi3l1 is highly expressed in epicardial adipose tissue and is associated with atrial fibrosis [143].

### 4.3. Blood

Blood diseases, such as acute myeloid leukemia (AML), sepsis, and acute lymphoblastic leukemia (ALL), are also classified as a series of circulatory system disorders. As a scientific challenge, sepsis can be diagnosed by increased Chi3l1 levels, while downregulated Chi3l1 levels have the potential to prevent skeletal muscle stem cell injury in sepsis [144,145]. Chi3l1 is also a diagnostic and prognostic biomarker for AML and ALL, and it is correlated with patient survival [146,147]. Additionally, high levels of Chi3l1 can be used to evaluate certain blood infection-related diseases, including endotoxemia and *Streptococcus pneumoniae* [148,149]. Notably, extracellular accumulation of the neutrophil-derived protein Chi3l1 significantly increases in a time-dependent manner during the storage of various blood components [150]. This finding indicates that the accumulation of Chi3l1 can be effectively prevented by prestorage leukocyte depletion through whole blood filtration, thus contributing to attenuating post-transfusion infectious complications [150].

## 5. Roles and Mechanisms of Chi3l1 in Diseases of the Nervous System

### 5.1. Neuron

Many studies have shown that Chi3l1 is expressed and secreted by various cell types of the nervous system, such as activated microglia and astrocytes, making it a potential biomarker for neurological diseases [151]. Many neuron-related inflammatory diseases, such as neurodegenerative disorders, are difficult to diagnose effectively. Reportedly, Chi3l1 is significantly increased and a promising biomarker for multiple neurodegenerative diseases, including Alzheimer’s disease (AD), Parkinson’s disease (PD), Huntington’s disease (HD), dementia, and amyotrophic lateral sclerosis (ALS) (Figure 1).

Exploring reliable diagnostic biomarkers for Alzheimer’s disease (AD) progression, especially in the early stages of AD, is highly important. Accumulating research has shown that Chi3l1 levels are greater in patients with early AD (407.81 ± 73.25 ng/mL) than in controls (96.91 ± 11.02 ng/mL) or patients with mild cognitive impairment (MCI; 176.49 ± 25.68 ng/mL), making it a potential biomarker for preclinical AD [152,153,154]. Notably, the differences in Chi3l1 levels between AD patients and healthy controls are mainly in plasma and cerebrospinal fluid rather than in serum, according to a recent meta-analysis [155]. *Chi3l1* gene polymorphisms (e.g., rs4950928 C>G and rs10399931 C>T) can affect the expression of Chi3l1 as well as the risk and prognosis of AD [156]. Another study revealed that Chi3l1 expression is regulated by the astrocyte circadian clock, which is reflected in the downregulation of Chi3l1 levels after the deletion of the core clock proteins BMAL1 or CLOCK/NPAS2, as well as its upregulation after deletion of the negative clock regulators PER1/PER2 [157]. Additionally, the expression of Chi3l1 is related to age and sex in AD patients [158]. Chi3l1 is significantly correlated with AD core biomarkers such as tau protein and amyloid beta (Aβ), whereas core biomarkers of neurodegeneration, such as tau protein, can accurately distinguish different neurodegenerative diseases, such as Alzheimer’s disease dementia and frontotemporal dementia [159,160]. A recent study revealed that CSF Chi3l1 can mediate Aβ-induced tau phosphorylation and tau-induced neuronal injury [161]. Considering the potential effects of polyunsaturated fatty acids (PUFAs) on AD, a study further revealed that peroral supplementation of omega-3 fatty acids can result in increasing the level of the cerebrospinal fluid biomarker Chi3l1 in patients with AD, indicating possible aggravation of the inflammatory response and axonal damage [162]. In the APP/PS1 mouse model of AD, *Chi3l1* deletion contributes to a decreased amyloid plaque burden and increased peri-plaque expression of the microglial lysosomal marker CD68 at the transcriptional level, indicating that Chi3l1 may suppress glial phagocytic activation and promote amyloid accumulation [157]. In a 5×FAD mouse model of AD, astrocyte-specific knockout of *Chi3l1* can reduce the amyloid plaque burden and restore memory functions [163].

Parkinson’s disease (PD) and Huntington’s disease (HD) are also important types of neurodegenerative diseases worldwide. Following AD, PD is the second most common neuropathological disorder, the progression of which closely correlates with mitochondrial dysfunction and inflammation [164]. Chi3l1 levels are threefold higher in PD patients than in controls [165]. There is a correlation between bioenergetic indices such as basal respiration or ATP production and Chi3l1 protein levels, which may be explained by the close relationship between inflammation and metabolism [165]. These findings suggest an interplay between Chi3l1 and mitochondrial function in PD, suggesting that the combination of Chi3l1 levels and changes in mitochondrial function might be used to more effectively evaluate inflammatory activity and the clinical course of PD. Additionally, increased Chi3l1 levels are detected in lipopolysaccharide (LPS)-induced PD model rats and are related to the release of inflammatory cytokines [166]. HD is an inherited neurodegenerative disorder caused by a CAG triplet repeat expansion on exon 1 in the huntingtin gene [167]. Reportedly, Chi3l1 levels are elevated both in the plasma and CSF of Huntington’s disease mouse models [168]. In patients with HD, Chi3l1 levels also tend to increase, further supporting its role as a reliable biomarker for diagnosing HD [169].

Dementia comprises a group of disorders that cause cognitive dysfunctions and daily life impediments in humans. It mainly includes neurodegenerative dementias and secondary dementias. Among them, dementia in AD, PD, and HD patients is an important type of neurodegenerative dementia, with AD being the most common cause of dementia [170]. Vascular dementia is the most common type of secondary dementia, and approximately 10–15% of patients are diagnosed with vascular-only dementia [170]. Chi3l1 has been demonstrated to be a pathophysiological biomarker of neurodegenerative dementia, contributing to the early diagnosis and prognosis of this disease [170,171]. In patients with vascular dementia, the level of the inflammatory marker Chi3l1 is significantly higher in *Helicobacter pylori* (Hp)-positive patients than in Hp-negative patients, suggesting that Hp-induced inflammation may be a risk factor for atherosclerosis in patients with vascular dementia [172].

Amyotrophic lateral sclerosis (ALS) is a fatal and progressive neurodegenerative disorder characterized by degeneration of both upper and lower motor neurons. A recent meta-analysis revealed that CSF Chi3l1 levels are significantly increased in ALS patients compared with healthy controls [173]. In ALS patients, Chi3l1 is significantly upregulated at the transcriptional and protein levels in monocyte-derived macrophages (MoMas) [174]. In addition, Chi3l1 is also expressed by activated astrocytes from the white matter of the motor cortex and the spinal cord in patients with ALS [175]. Chi3l1 levels are associated with the rate of ALS progression, with higher CSF Chi3l1 levels observed in fast-progressing disease than in slow-progressing disease [175].

### 5.2. Brain

Many studies have shown that Chi3l1 is widely expressed in all regions of the human brain and serves as a diagnostic biomarker and therapeutic target for multiple brain-related inflammatory diseases (e.g., encephalitis, psychosis, multiple sclerosis, and brain injury) (Figure 1).

Encephalitis, such as autoimmune encephalitis and viral encephalitis, is potentially lethal and affects mainly children. To improve treatment and patient outcomes, effective biomarkers can be used to monitor and predict the prognosis of this disease. In the case of autoimmune encephalitis, cerebrospinal fluid Chi3l1 produced by glial cells is closely correlated with the clinical course of this disease [176]. Specifically, cerebrospinal fluid levels of Chi3l1 are increased in both anti-gamma-aminobutyric-acid-B receptor (GABAbR) encephalitis and anti-N-methyl-D-aspartate receptor (NMDAR) encephalitis [177,178]. In addition, Chi3l1 is a promising biomarker for various types of viral encephalitis, such as tick-borne encephalitis (TBE) and human immunodeficiency virus encephalitis (HIVE) [179,180]. During HIVE, Chi3l1 can interfere with the biological activity of basic fibroblast growth factor to affect neuronal function or survival [181]. Additionally, impaired autophagy may lead to abnormal protein aggregation during central nervous system infections (e.g., encephalitis and meningitis); thus, both Chi3l1 and lysosome-associated membrane proteins (LAMPs) hold promise as biomarkers or therapeutic targets for these neurological diseases [182].

Psychosis refers to a mental state of dysfunction in behavior and thought processes, and many related diseases correlate with aberrant structural and functional abnormalities in the brain. Reportedly, Chi3l1 can act as a potential biomarker for certain psychoses, thereby assisting in the diagnosis of these diseases. In patients with bipolar disorder (BD), a reduction in BD-related brain subregion volume is related to increased plasma levels of Chi3l1, whereas macrophages and macrophage-like cells may be involved in brain volume reduction [183]. A recent study suggested a weak but positive correlation between serum Chi3l1 levels and cognitive functions in patients with bipolar disorder [184]. Schizophrenia is a common and complex psychiatric disease in which Chi3l1 levels are increased in the hippocampus and prefrontal cortex regions of patients [185]. According to a multicenter case–control study and meta-analysis, genetic variants within the *Chi3l1* gene exhibit ethnic heterogeneity and confer susceptibility to schizophrenia among Asian populations [185]. Moreover, transcript expression of the schizophrenia susceptibility gene *Chi3l1* is regulated by cis-variation in lymphoblasts, while polymorphisms within the promoter region of *Chi3l1* significantly correlate with this allelic imbalance [186]. Additionally, Chi3l1 is a prognostic biomarker for clinically isolated syndromes (CISs), showing high expression levels among CIS patients with the conversion to multiple sclerosis compared with those continued as CIS [187]. Chi3l1 can also serve as a biomarker in other psychoses, such as delirium, drug-resistant epilepsy, and first-episode psychosis [188,189,190].

Multiple sclerosis (MS) is a chronic inflammatory disease affecting the central nervous system, such as the brain and spinal cord. Multiple studies have confirmed that Chi3l1 can act as a diagnostic and prognostic biomarker for MS and predict the conversion from clinically isolated syndrome (CIS) to MS [191,192]. Chi3l1 is expressed in MS-related microglia and macrophages as well as in the astrocytes of MS brains [193]. High levels of CSF Chi3l1 are associated with increased disability, including motor, cognitive, and radiological aspects, in patients with multiple sclerosis [194]. Because CSF Chi3l1 is correlated with spinal cord volume loss, whereas CSF neurofilament light chain (NfL) is related to gray matter atrophy, these two biomarkers can provide complementary information for determining the course and prognosis of MS [193]. Moreover, their combined measurement contributes to discriminating MS phenotypes, including primary progressive MS (PPMS), secondary progressive MS (SPMS), and relapsing remitting MS (RRMS) [195]. Additionally, the CSF Chi3l1/Chi3l2 ratio can accurately distinguish progressive MS (PMS) from RRMS [191]. Chi3l1 levels are significantly increased in PMS patients compared with RRMS patients but remain similar between RRMS patients during the relapse and remission periods [194,196]. Furthermore, gene polymorphisms of *Chi3l1*, such as allele C of rs4950928, are significantly correlated with PPMS patients [196]. Mechanistically, Chi3l1 can exert cytotoxic effects by inducing neurite length retraction and reducing neuronal survival only in neurons but not in other central nervous system cells, indicating that Chi3l1 is a potential therapeutic target for MS patients [197].

Some other inflammatory brain diseases also exhibit a close correlation with Chi3l1 levels. Traumatic brain injury (TBI) is a critical cause of death and disability. Accumulating evidence has shown that Chi3l1 is a promising biomarker for determining the presence, location, and extent of TBI [198]. Intracerebral hemorrhage (ICH) can damage the brain parenchyma, and Chi3l1 is an effective biomarker for evaluating the severity and predicting long-term clinical outcomes of ICH [199]. Moreover, the serum Chi3l1 level is also a candidate prognostic biomarker for cerebral amyloid angiopathy-related intracerebral hemorrhage recurrence [200]. High Chi3l1 levels in both cerebrospinal fluid and serum are also detected in patients with aneurysmal subarachnoid hemorrhage [201]. Additionally, in autoimmune brain diseases such as neuromyelitis optica (NMO), Chi3l1 reportedly impairs hippocampal neurogenesis and cognitive function, whereas blocking the Chi3l1/CRTH2/β-catenin cascade contributes to restoring neurogenesis and improving cognitive deficits (Figure 2) [202].

## 6. Roles and Mechanisms of Chi3l1 in Diseases of the Urinary System

Kidney- and bladder-related inflammatory diseases have been reported to be significantly correlated with high Chi3l1 concentrations (Figure 1). In a mouse model of renal atrophy, increased expression of Chi3l1 mRNA was detected in infiltrating macrophages and neutrophils [203]. Urine or plasma Chi3l1 can act as valuable noninvasive biomarkers for acute kidney injury (AKI); for example, the levels of urinary Chi3l1 in patients with stage Ι AKI (9.13 ± 1.22 ng/mL), stage ΙΙ AKI (11.30 ± 1.54 ng/mL), and stage ΙΙΙ AKI (13.13± 1.16 ng/mL) continue to increase [204,205,206,207]. Chronic kidney disease (CKD) is associated with increased development of cardiovascular complications, and plasma Chi3l1 levels increase with CKD stage [208]. End-stage kidney disease (ESKD) represents the continuous progression of CKD characterized by an increased cardiovascular mortality rate, and dialysis and kidney transplantation are critical and effective therapies for ESKD. There are sex-specific relationships between Chi3l1 levels and cardiovascular complications in ESKD patients [209]. For example, increased Chi3l1 levels are associated with vascular calcification, inflammation, oxidative stress, and all-cause mortality only in males but not in females [209]. In chronic hemodialysis patients, Chi3l1 levels are positively correlated with multiple factors, such as cardiovascular events, abdominal aortic calcification, autologous arteriovenous fistula (AVF) failure, gamma-glutamyl transpeptidase (gamma-GTP) levels, the geriatric nutritional risk index (GNRI), and age [210,211,212,213]. Additionally, increased Chi3l1 levels are independently associated with proteinuria, cardiovascular disease, and endothelial dysfunction in renal transplant recipients [214,215]. Moreover, urinary Chi3l1 has the potential to determine the suitability of donor kidneys for transplantation [216]. In addition to the kidney, bladder-related inflammatory diseases also pose a severe threat to human health and can be evaluated by Chi3l1 levels. For example, bladder pain syndrome/interstitial cystitis (BPS/IC) is a chronic disease affecting the urinary bladder that may lead to detrusor fibrosis. Chi3l1 is expressed in the detrusor mast cell granules and submucosal macrophages of BPS/IC patients and is associated with detrusor fibrosis, indicating that the serum and urine levels of Chi3l1 can serve as valuable biomarkers for evaluating bladder fibrogenesis in this disease [217].

## 7. Roles and Mechanisms of Chi3l1 in Diseases of the Endocrine System

Effective diagnostic methods for endocrine system-related diseases such as diabetes, obesity, insulin resistance, and Graves’ disease are essential, and Chi3l1 is widely reported to be a reliable biomarker (Figure 1).

Diabetes mellitus (DM) is a common disease worldwide and can be classified into type 1 diabetes mellitus (T1DM), type 2 diabetes mellitus (T2DM), and gestational diabetes mellitus (GDM). Numerous studies have demonstrated that Chi3l1 levels are increased in DM patients and that it may be produced by neutrophils and associated with a severe degree of albuminuria [218,219]. In T2DM patients, Chi3l1 is also correlated with metabolic syndrome, dyslipidemia, and glycemic parameters such as HbA1c, albuminuria, and fasting glucose [5]. In GDM patients, Chi3l1 levels are correlated with glycated hemoglobin, fasting insulin, and homeostasis model assessment of insulin resistance (HOMA-IR) [220]. DM can cause injury to multiple organs due to persistent hyperglycemia, thereby leading to a series of diabetic complications [221]. Diabetic nephropathy (DN) is one of the most serious complications of DM, and Chi3l1 levels are closely associated with kidney function decline and mortality in both T1DM and T2DM patients [222,223,224]. Another severe DM complication is diabetic foot, which is characterized by delayed wound healing. Higher Chi3l1 levels are detected in diabetic foot cases (140.2 ± 125.3 ng/mL) than in control groups (26.9 ± 13.9 ng/mL), while STAT3 upregulation contributes to wound healing by promoting the proliferation and migration of fibroblasts via targeting Chi3l1/MAPK signaling (Figure 2) [225,226]. The metabolic abnormalities caused by DM can lead to a series of cardiovascular complications, and Chi3l1 can be an effective diagnostic marker and potential therapeutic target for vascular complications in patients with both T1DM and T2DM [227,228]. Reportedly, Chi3l1 can predict coronary artery disease (CAD) in asymptomatic patients with T2DM, and the single nucleotide polymorphism (SNP) rs946263 of the *Chi3l1* gene is correlated with both insulin resistance and the severity of CAD in T2DM patients [229,230]. Additionally, a relationship (*r* = −0.508, *p* < 0.01) between increased Chi3l1 levels and decreased miR-24 levels has been detected in T2DM patients with coronary heart disease (CHD), and miR-24 functions to regulate the expression of the conserved target Chi3l1 by binding to the 3′ UTR of Chi3l1 mRNA [231]. Higher Chi3l1 levels are also observed in other DM-associated cardiovascular complications, such as ischemic heart disease (IHD) and peripheral arterial disease (PAD) [232,233]. Diabetic retinopathy (DR) is a microvascular complication of diabetes characterized by retinal vascular microaneurysm and blot hemorrhages [221]. Chi3l1 levels are significantly increased in patients with DR [234]. Further studies indicate that Chi3l1 is correlated with morphological parameters of retinal blood vessels (e.g., diameter and number) in DR patients [235].

Obesity is widely regarded as the initiation and establishment of a low-grade inflammatory state characterized by the presence of numerous circulating inflammatory molecules, which may further lead to insulin resistance (IR) and even diabetes [221]. Chi3l1 levels increase in morbidly obese patients but decrease after weight loss [236]. Moreover, Chi3l1 is associated with homeostasis model assessment of insulin resistance (HOMA-IR) (*R* = 0.604, *p* = 0.029), fasting insulin levels (*R* = 0.622, *p* = 0.023), and monocyte chemoattractant protein-1 (MCP-1) (*R* = 0.805, *p* = 0.001), indicating its potential roles in the development of IR, T2DM, and even cardiovascular mortality in obese patients [236]. In obese prepubertal children, reduced ghrelin levels, rather than elevated leptin levels, result in Chi3l1 upregulation [237]. In obesity-associated T2DM patients, both circulating and visceral adipose tissue expression levels of Chi3l1 significantly increase, whereas upregulated Chi3l1 levels can be decreased through weight loss resulting from a conventional hypocaloric diet but not gastric bypass surgery [238]. Finally, Graves’ disease (GD), an autoimmune thyroid disease, is associated with increased levels of Chi3l1 [239]. Chi3l1 is correlated with GD severity and may be involved in the pathogenesis of GD [239].

## 8. Roles and Mechanisms of Chi3l1 in Diseases of the Skeletal System

### 8.1. Joints

Joints serve as critical parts of the skeletal system, and their associated inflammatory diseases (e.g., rheumatoid arthritis, osteoarthritis, and psoriatic arthritis) seriously affect human health and quality of life. Thus, identifying potential diagnostic biomarkers and therapeutic targets is highly important, and Chi3l1 is a promising protein (Figure 1).

As a chronic autoimmune inflammatory disease, rheumatoid arthritis (RA) affects mainly the synovial joints and is characterized by synovial inflammation, joint swelling, and cartilage degradation. Numerous studies have demonstrated that Chi3l1 levels are higher in RA patients than in healthy individuals [240]. Reportedly, Chi3l1 is derived mainly from synovial cells, chondrocytes, and neutrophils in the arthritic joint, with its mRNA expression increasing after 2 and 4 weeks of joint immobility [241,242]. Promoter polymorphisms are known to significantly influence serum Chi3l1 levels both in RA patients and healthy subjects; however, there is no significant association between functional variants of Chi3l1 and RA disease [243,244]. In patients with RA, Chi3l1 levels closely correlate with rheumatoid factor (RF) levels, RA activity (e.g., 28-joint disease activity score (DAS28), erythrocyte sedimentation rate (ESR), and C-reactive protein (CRP) level), proinflammatory cytokines (e.g., tumor necrosis factor-α (TNF-α) and interleukin-1B (IL-1β)), and immunological markers of joint destruction such as matrix metalloproteinase-3 (MMP-3) [240,245,246]. Additionally, RA can cause extra-articular manifestations such as interstitial lung disease (ILD), which is associated with poor prognosis and increased mortality in RA patients. Furthermore, Chi3l1 levels are significantly increased in patients with RA-ILD, suggesting its possible role as a biomarker to detect RA-ILD noninvasively [247]. According to a previous report, arg-vasopressin (AVP) and parathyroid hormone-related protein (PTHrP) promote cell proliferation and Chi3l1 secretion in human chondrocytes derived from RA patients [248]. Angiogenesis is considered a crucial step in the pathogenesis of RA, and Chi3l1 can induce interleukin-18 (IL-18) expression in osteoblasts and promote angiogenesis in endothelial progenitor cells (EPCs) by inhibiting miR-590-3p via the focal adhesion kinase (FAK)/PI3K/Akt cascade (Figure 2) [249].

Chi3l1 has been widely demonstrated to be a potential biomarker for the severity of osteoarthritis (OA) [250]. Moreover, combined detection of Chi3l1/collagen type II (CTX-II) or Chi3l1/ultrasonography can provide more sensitive and reliable information for diagnosing OA [251,252]. Cartilage destruction of matrix metalloproteinases (MMPs) and proinflammatory cytokines such as interleukin-6 (IL-6) and interleukin-17 (IL-17) are involved in the pathogenesis of OA. Previous studies have reported that cartilage acts as a main source of Chi3l1 in OA joints and that IL-6 and IL-17 promote the expression of Chi3l1 in primary OA chondrocytes [253]. Additionally, Chi3l1 levels are positively correlated with MMP-1, MMP-3, IL-6, and IL-17 levels [253]. Partially acetylated chitooligosaccharides can bind to Chi3l1 and stimulate the growth of osteoarthritic chondrocytes, providing a critical theoretical foundation for OA therapy [254]. In a post-traumatic osteoarthritis (PTOA) mouse model, the levels of Chi3l1 and several inflammatory cytokines, such as TNF-α, IL-1β, and IL-6, significantly decrease in nuclear factor erythroid 2-related factor 2 (Nrf2)-overexpressing mice [255]. These findings indicate that Nrf2 can effectively attenuate inflammation by negatively regulating Chi3l1, thus further improving post-traumatic osteoarthritis [255].

Several other joint inflammatory diseases, such as psoriatic arthritis (PsA) and juvenile idiopathic arthritis (JIA), are also diagnosed on the basis of Chi3l1 levels. PsA is a type of arthropathy characterized by inflammatory joint changes and psoriasis. Chi3l1 levels are largely elevated in patients with psoriatic arthritis but not in those with psoriasis [256]. Chi3l1 serum levels are significantly correlated with the 28-joint disease activity score (DAS 28), body surface area (BSA), and psoriasis area and severity index (PASI), thereby contributing to diagnosing and monitoring the severity of joint involvement in patients with psoriatic arthritis [257]. As universally recognized and effective treatments for PsA patients, tumor necrosis factor α (TNF-α) inhibitors significantly decrease the serum concentrations of Chi3l1, IL-6, and MMP-1, confirming the usefulness of these mediators in monitoring the effectiveness of anti-TNF-α treatment [258]. JIA is a common type of inflammatory arthropathy in pediatric patients. Chi3l1 levels are reportedly increased in JIA patients, making it a useful biomarker for disease activity and therapeutic efficacy [259,260].

### 8.2. Bone

Bone abnormalities also trigger a series of inflammatory diseases related to the skeletal system, including osteoporosis, spondylitis, intervertebral disc degeneration, and osteomyelitis. The effective diagnosis of these diseases depends on reliable biomarkers, and Chi3l1 is one such indicator (Figure 1).

Osteoporosis is a common systemic skeletal disease characterized by bone fragility. Chi3l1 levels are increased in osteoporosis, which is regulated by enhancing METTL3-mediated m6A methylation of Chi3l1. Furthermore, early growth response 1 (EGR1), a transcription factor of METTL3, can promote osteoclast differentiation and osteoporosis development by regulating the METTL3/m6A/Chi3l1 axis [261]. Additionally, Chi3l1 also induces osteoblast differentiation by promoting bone morphogenetic protein 2 (BMP2) signaling and suppresses osteoclastogenesis by increasing osteoprotegerin (OPG) via noncanonical BMP2 signaling (Figure 2) [262]. In patients with spondylitis, higher Chi3l1 levels are accompanied by higher disease activity scores, indicating that Chi3l1 can be used as a useful biomarker for the early diagnosis of this disease [263]. In addition, treatment with TNFα inhibitors can change Chi3l1 levels in spondylitis patients, further confirming the utility of this protein in monitoring therapeutic efficacy [264]. Intervertebral disc degeneration (IDD) easily causes low back pain and may even result in disability. Comprehensive network analysis has identified Chi3l1 as a novel biomarker for IDD, and its relationship with inflammatory substances such as cyclooxygenase-2 (COX-2) and nitric oxide (NO) has been confirmed in disc tissue culture [265,266]. The main pathophysiological process of IDD involves imbalances between the synthesis and degradation of the extracellular matrix (ECM) in nucleus pulposus (NP) cells [267]. Chi3l1 is highly expressed by NP cells in IDD, which significantly decreases catabolism and increases the anabolism of the ECM [268]. A further study revealed that Chi3l1 protects the nucleus pulposus via AKT3 signaling during IDD (Figure 2) [268]. Another study revealed that Chi3l1 secreted by M2a macrophages can promote imbalances in extracellular matrix metabolism through the IL-13Rα2 receptor and activation of the ERK and JNK MAPK cascades in IDD (Figure 2) [269]. Osteomyelitis is an infectious inflammatory disease of the bone, that is mainly caused by invasion from *Staphylococcus aureus* (*S. aureus*). Chi3l1 can aggravate *S. aureus*-induced osteomyelitis by mediating osteoblast differentiation and proliferation through the activation of the p38/MAPK and Smad pathways, whereas the inhibition of Chi3l1 contributes to reducing the debilitating effects of *S. aureus* in this disease (Figure 2) [270,271].

## 9. Roles and Mechanisms of Chi3l1 in Diseases of the Muscular System

Exploring novel biomarkers for diagnosing early inflammatory muscle diseases contributes to improving patient prognosis, and Chi3l1 has been reported to be a promising marker and potential target for these disorders (Figure 1) [272]. Idiopathic inflammatory myopathy (IIM), also known as myositis, is correlated with increased levels of Chi3l1, which potentially predicts myocardial injury in IIM patients [273]. Specifically, higher serum levels of Chi3l1 were detected in the myocardial injury group than in the non-myocardial injury group (79.4 (62.7, 213.8) ng/mL vs. 44.4 (18.0, 59.3) ng/mL, *p* < 0.001) [273]. Both polymyositis (PM) and dermatomyositis (DM) are inflammatory muscle diseases characterized by muscle weakness, and Chi3l1 levels are significantly increased in PM/DM patients [274]. A cross-sectional study has been performed to investigate the clinical value of Chi3l1 in patients with PM/DM, which suggests that the serum Chi3l1 level is a possible useful biomarker for PM/DM diagnosis [272]. Moreover, the infiltration of Chi3l1-positive macrophages into the intramuscular sheath and perimuscular membrane is also detected in the pathogenesis of myositis, and the mechanism by which Chi3l1-positive macrophages contribute to myositis will be a subject of future research [274]. Antisynthetase syndrome (ASSD) is a rare systemic autoimmune myopathy. Chi3l1 is highly expressed by inflammatory cells in the muscle tissue of ASSD patients compared with healthy individuals (538.4 (363.4–853.1) pg/mL versus 270.0 (201.8–451.9) pg/mL, respectively; *p* < 0.001), and it is associated with TNF-α levels (Spearman’s correlation, *rho* = 0.382; *p* = 0.007) [275]. In patients with muscle atrophy and weakness induced by sepsis, elevated levels of Chi3l1 correlate with disease severity [276]. Chi3l1 overexpression significantly increases the levels of IL-1β and Caspase1 in muscle satellite cells, whereas this effect is reversed when Chi3l1 is silenced [276]. STAT6 inhibition can mitigate sepsis-induced muscle weakness by downregulating Chi3l1 levels and modulating mitochondrial dysfunction and ferroptosis (Figure 2) [276].

## 10. Roles and Mechanisms of Chi3l1 in Diseases of the Reproductive System

Many studies have shown that Chi3l1 serves as a useful biomarker for a series of reproductive diseases (Figure 1). Among them, most studies are associated with female reproductive diseases, while few studies have been conducted in male groups. Endometriosis is a common reproductive disease among women and can be diagnosed and evaluated for severity on the basis of increased levels of Chi3l1 [277,278]. A positive correlation between serum Chi3l1 levels and the stage of endometriosis has been revealed, and the inflammatory process during endometriosis may result in elevated Chi3l1 levels [277,278]. Further studies suggest that a triple combination panel of Chi3l1/IL-37/CA125 and a quadruple combination panel of Chi3l1/CA125/endocan/copeptin can provide more sensitive and specific diagnoses of the endometriosis stage, especially moderate-to-severe endometriosis [279,280]. Polycystic ovary syndrome (PCOS), another female reproductive disease, can be characterized by increased Chi3l1 levels [281,282]. Furthermore, there is a significant difference in Chi3l1 levels between women with PCOS and abnormal glucose tolerance (AGT-PCOS; 169.14 ± 36.1 μg/L) compared with women with PCOS and normal glucose tolerance (NGT-PCOS; 147.7 ± 41.9 μg/L), which may be beneficial in predicting abnormal glucose tolerance in patients with PCOS [281]. Moreover, Chi3l1 plays an important role in insulin resistance and diabetes, which closely correlates with glypican-4 (GPC-4), neuregulin-4 (NRG4), body mass index (BMI), the waist-to-hip ratio (WHR), and homeostasis model assessment of insulin resistance (HOMA-IR) in women with PCOS [282]. Oxidative stress is involved in the pathogenesis of PCOS. A mechanistic study suggested that Chi3l1 expression is induced by oxidative stress, while Chi3l1 knockdown decreases oxidative stress damage by activating the PI3K/AKT pathway and suppressing NF-κB signaling in ovarian granulosa cells (Figure 2) [283]. Preeclampsia is a disease associated with the maternal inflammatory response. In patients with preeclampsia, elevated levels of Chi3l1 are related to disease severity, which is verified by the difference in Chi3l1 levels in patients with more severe proteinuria and milder proteinuria (median 121.3 ng/mL versus median 55.4 ng/mL; *p* = 0.022) [284]. Another study reported that serum Chi3l1 levels increase in the early stage of gestation and then decrease in the late stage. However, elevated Chi3l1 levels are not associated with preeclampsia but are related to maternal age, body mass index, and small for gestational age [285]. Additionally, Chi3l1 is highly expressed in placenta percreta and is correlated with extravillous trophoblast invasion [286]. Upregulated Chi3l1 can act as a diagnostic and prognostic biomarker for placenta accreta spectrum disorders (PASs), and a mechanistic study further revealed that Chi3l1 significantly promotes the proliferation and invasion of HTR-8/SVneo cells via the activation of Akt/MMP9 signaling in this disease (Figure 2) [287,288]. In addition, Chi3l1 levels are significantly increased in patients with pelvic inflammatory disease and contribute to the prediction of disease severity [289]. There is also a correlation between Chi3l1 levels and preterm birth according to a mouse model study [290]. Finally, few studies have explored the roles of Chi3l1 in male reproductive diseases. For example, endothelial dysfunction may function in the pathogenesis of male idiopathic infertility, while Chi3l1 levels are increased and act as markers for endothelial dysfunction in male patients with idiopathic infertility [291]. Furthermore, serum levels of Chi3l1 are positively correlated with the duration of infertility and the serum level of FSH [291]. Seminal Chi3l1 levels are increased in infertile oligoasthenoteratozoospermic (OAT) men with varicocele, and protein levels are negatively correlated with sperm concentration, total sperm motility, and normal sperm morphology [292]. Following varicocelectomies, seminal Chi3l1 levels tend to decrease [292]. Overall, Chi3l1 is a potential therapeutic target for reproductive system-related inflammatory diseases.

## 11. Therapeutic Approaches Involving Chi3l1 for Multiple Human Body System Diseases

**Figure 3:**
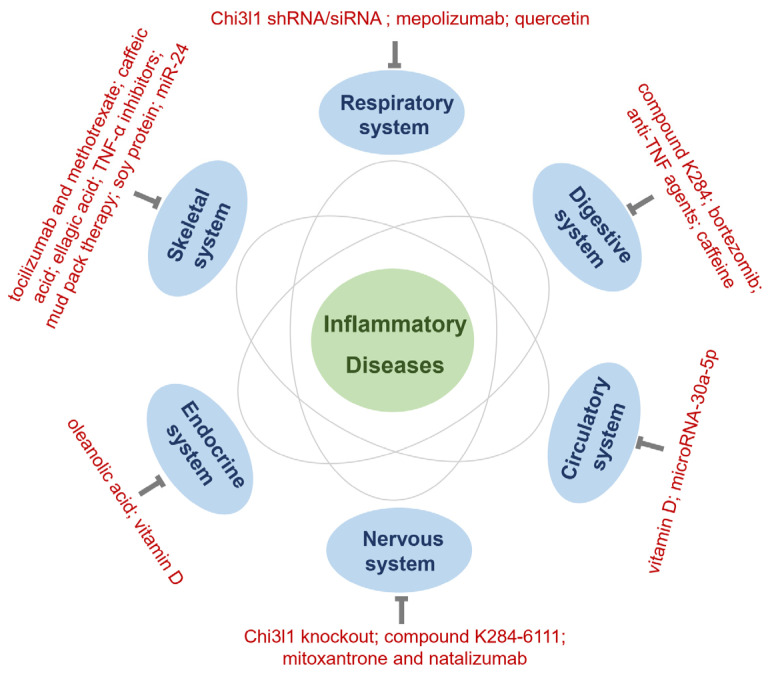
Summary of therapeutic strategies involving Chi3l1 for multiple human body system diseases. Various therapeutic methods are used to alleviate inflammatory diseases of the respiratory, digestive, circulatory, skeletal, nervous, and endocrine systems by downregulating Chi3l1 levels.

Given the aforementioned roles and mechanisms of Chi3l1 in chronic inflammation related to multiple body systems (Figure 1 and Figure 2), an increasing number of studies have verified Chi3l1 as a promising therapeutic target. Many studies have demonstrated that regulating Chi3l1 expression or relevant signaling pathways can effectively alleviate inflammatory diseases in multiple organ systems, such as the respiratory, digestive, circulatory, skeletal, nervous, and endocrine systems (Figure 3). In respiratory system-associated inflammatory diseases, RNA interference, antibodies, and drugs can attenuate inflammatory responses by regulating Chi3l1 expression. For example, adenovirus vector-mediated Chi3l1 shRNA can downregulate Chi3l1 levels and alleviate eosinophilic airway inflammation, airway hyperresponsiveness, and airway mucus secretion in an asthmatic mouse model, which highlights treatment prospects for human asthma [293]. Moreover, bone marrow signaling molecules including eotaxin, granulocyte-macrophage colony-stimulating factor (GM-CSF), and interleukin (IL)-5 significantly decrease and correlate with decreased levels of Chi3l1, indicating that Chi3l1 could be involved in asthma inflammation by altering bone marrow signaling molecules [293]. Chi3l1 siRNA also decreases the expression of eosinophilic airway inflammation-related factors, including IL-5, eotaxin, and GM-CSF, at the mRNA and protein levels in an epithelial cell model of asthma, thus reducing airway inflammation [294]. Another study reported that treatment with a humanized anti-IL-5 monoclonal antibody called mepolizumab (MEP) can attenuate allergen-induced Th2 inflammation and tissue remodeling by downregulating Chi3l1 levels in asthma patients [295]. Quercetin relieves acute lung injury by decreasing Chi3l1 and oxidant molecule levels (xanthine oxidase (XO), nitric oxide (NO), and malondialdehyde (MDA); *p* < 0.05), as well as increasing antioxidant enzyme levels (superoxide dismutase (SOD) and catalase (CAT); *p* < 0.05) [296]. Furthermore, quercetin decreases the serum levels of Chi3l1 and periostin in cecal ligation and puncture-induced acute lung injury (ALI) rats, suggesting that quercetin may be a useful therapeutic reagent for sepsis-induced ALI [296]. For digestive system diseases, the compound K284 alleviates lipopolysaccharide (LPS)-induced acute liver injury by inhibiting the expression of Chi3l1 and CXCL3 [68]. Bortezomib effectively attenuates steatotic liver ischemia/reperfusion injury by suppressing the expression of MMP and Chi3l1 [73]. Anti-TNF agent-mediated Chi3l1 downregulation can ameliorate Crohn’s disease by restricting the replication of adherent-invasive *Escherichia coli* (AIEC) within macrophages [297]. Caffeine treatment is able to mitigate acute colitis by inhibiting Chi3l1 levels, reducing bacterial invasion, and deactivating the AKT cascade [298]. With respect to circulatory system-related diseases, the progression of cardiovascular disease (CVD) can be prevented by vitamin D-mediated Chi3l1 suppression, and this suppression may be achieved by inhibiting IL-6, soluble intercellular adhesion molecule-1 (sICAM-1), and soluble vascular cell adhesion molecule-1 (sVCAM-1) [299]. MicroRNA-30a-5p promotes proliferation and inhibits the apoptosis of human pulmonary artery endothelial cells by downregulating Chi3l1 levels through binding to the 3′-UTR of Chi3l1; thus, the miR-30a-5p/Chi3l1 axis may be a potential target in the treatment of pulmonary arterial hypertension (PAH) [300]. In nervous system diseases, the ERK and NF-κB signaling pathways involved in neuroinflammation are associated with the development and progression of Alzheimer’s disease (AD) [301,302]. Pentraxin-3 (PTX3) can be regulated by ERK signals, whereas PTX3 can regulate NF-κB signals [301,302]. Chi3l1 deficiency can ameliorate AD by reducing the amyloid-beta burden, restoring memory functions, and inhibiting neuroinflammation via blockade of the ERK-dependent PTX3 pathway [163,301]. Some drugs, such as the Chi3l1 inhibitor K284-6111, can attenuate memory impairment and neuroinflammation by modulating the NF-κB and ERK-PTX3 pathways, thus potentially preventing AD development [302,303]. Additionally, immunosuppressive treatments such as mitoxantrone and natalizumab play potential roles in multiple sclerosis (MS) therapy by decreasing cerebrospinal fluid (CSF) levels of Chi3l1 [304]. Chi3l1 and its downstream mitogen-activated protein kinase (MAPK) pathway are reported to be correlated with wound healing in diabetic foot ulcers (DFUs) [226]. For endocrine system diseases such as DFU, signal transducer and activator of transcription 3 (STAT3) can promote fibroblast proliferation and migration to facilitate wound healing by activating the Chi3l1/MAPK axis, providing drug targets for the treatment of this disease [226]. Oleanolic acid (OA) administration partially decreases the level of Chi3l1 from 14.60 ± 1.00 ng/mL to 10.60 ± 0.90 ng/mL and alleviates renal damage-related inflammatory and oxidative profiles in diabetic rats, indicating its potential application in diabetes and diabetic nephropathy [305]. Both Chi3l1 and monocyte chemoattractant protein-1 (MCP-1) are involved in inflammatory responses, which are positively associated with the progression of diabetes and its complications [306]. Compared with the placebo, vitamin D may contribute to diminishing vascular diabetic complications by reducing Chi3l1 levels (95.9 vs. 70.3 ng/mL, *p* = 0.003) and MCP-1 levels (241.2 vs. 179.0 pg/mL, *p* = 0.02) [306]. With respect to inflammatory diseases associated with the skeletal system, combined therapy with tocilizumab (TCZ) and methotrexate (MTX) can result in a significant increase in body mass index (BMI), total cholesterol (TC), high-density lipoprotein cholesterol (HDL-C), and triglyceride (TG) levels, and a significant decrease in the TC/HDL-C ratio and Chi3l1 levels in RA patients [307]. Caffeic acid, ellagic acid, and TNF-α inhibitors can ameliorate arthritis by targeting Chi3l1 [258,308]. Furthermore, caffeic acid and ellagic acid have similar effects on alleviating adjuvant-induced arthritis [308]. Additionally, mud pack therapy and soy protein can regulate Chi3l1 levels and have potential applications in the treatment of osteoarthritis [309,310]. MicroRNAs (miRNAs) have been shown to play a regulatory role in osteogenesis, and miR-24 inhibits the expression of Chi3l1 mRNA by binding to the 3′-untranslated region to attenuate the effects of *S. aureus* in osteomyelitis [311]. In the muscular system, Chi3l1 protects skeletal muscle from TNFα-induced inflammation and insulin resistance via a protease-activated receptor 2 (PAR2)-dependent mechanism, and further study has shown that acute exercise contributes to an increase in Chi3l1 levels from 19.5 ± 1.1 ng/mL to 22.8 ± 1.2 ng/mL and to human myocyte proliferation [312,313]. In the reproductive system, Chi3l1 can regulate TNFα-induced activation of uterine smooth muscle cells via the PAR2 pathway, potentially providing a therapeutic method for reducing preterm birth rates [314]. Overall, Chi3l1 is a potential therapeutic target for multiple human body system-related inflammatory diseases, and more relevant research needs to be conducted in the future.

## 12. Conclusions

Chi3l1 plays a critical role in the diagnosis and pathogenesis of multiple organ system-related inflammatory diseases. As described above, elevated Chi3l1 levels are detected in many types of diseases and are closely correlated with disease activity, severity, and prognosis. Many signaling pathways are also involved in the development of inflammatory diseases mediated by Chi3l1. The inhibition of Chi3l1 expression and regulation of relevant signaling cascades may provide promising therapeutic targets for multiple inflammatory disorders. Interestingly, Chi3l1 also contributes to ameliorating the progression of certain diseases. Although the inflammatory effects and regulatory mechanisms of Chi3l1 in many diseases have been clarified to a large extent, studies on its roles and mechanisms in specific diseases are lacking. This strongly limits the exploration of novel prevention and treatment strategies for these diseases. In addition, there are also the limitations of Chi3l1 as a biomarker due to the insufficient specificity and sensitivity, different inclusion criteria in the study, and differences in expression levels among individuals, etc. Thus, combined diagnosis of multiple biomarkers (e.g., noninvasive diagnosis of the endometriosis stage with a triple combination panel of Chi3l1/IL-37/CA125) could be a promising strategy for improving the accuracy of disease diagnosis. Notably, the current studies on Chi3l1 in inflammatory diseases are restricted to various disease models or clinical investigations, whereas reports concerning the clinical application of Chi3l1 for disease detection or treatment are quite scarce. Overall, this review not only provides a systematic and comprehensive understanding of the inflammatory effects and regulatory mechanisms of Chi3l1 in almost all human organ systems but also highlights current research deficiencies.

## Data Availability

Not applicable.
